# Insights into the Thermal Degradation Kinetics of β-Lactam Antibiotics: A Comparative Study of Cefazolin, Ceftriaxone, and Meropenem

**DOI:** 10.3390/antibiotics15060540

**Published:** 2026-05-27

**Authors:** Ovidiu Ghirlea, Amalia Ridichie, Mirela Voicu, Adriana Ledeți, Ioan Bîtcan, Laura Sbârcea, Diana Dreavă, Ionuț Ledeți, Cristina Trandafirescu, Marius Murariu

**Affiliations:** 1 Faculty of Medicine, “Victor Babeș” University of Medicine and Pharmacy, Eftimie Murgu Square No. 2, 300041 Timisoara, Romania; ovidiu.ghirlea@umft.ro (O.G.); murariu.marius@umft.ro (M.M.); 2 Advanced Instrumental Screening Center, Faculty of Pharmacy, “Victor Babeș” University of Medicine and Pharmacy, Eftimie Murgu Square No. 2, 300041 Timisoara, Romania; amalia.ridichie@umft.ro (A.R.); sbarcea.laura@umft.ro (L.S.); ionut.ledeti@umft.ro (I.L.); 3 Faculty of Pharmacy, “Victor Babeș” University of Medicine and Pharmacy, Eftimie Murgu Square No. 2, 300041 Timisoara, Romania; trandafirescu.cristina@umft.ro; 4 Faculty of Industrial Chemistry and Environmental Engineering, University Politehnica Timișoara, Vasile Pârvan 6, 300001 Timisoara, Romania; ioan.bitcan@upt.ro (I.B.); diana.dreava@upt.ro (D.D.)

**Keywords:** β-lactam antibiotics, isoconversional, ceftriaxone sodium, cefazolin sodium, meropenem, isokinetic temperature, thermal stability

## Abstract

**Background/Objectives**: The thermal stability and degradation kinetics of β-lactam antibiotics are critical for understanding their behavior under processing and storage conditions. This study investigates the thermal decomposition of meropenem, ceftriaxone sodium, and cefazolin sodium in order to evaluate their kinetic parameters, assess the presence of the compensation effect, and determine isokinetic temperatures. **Methods**: Thermal analysis was performed using simultaneous TG/DTG/DSC measurements. Non-isothermal degradation experiments were conducted at four different heating rates. Kinetic parameters were evaluated using two isoconversional methods (Friedman and Flynn–Wall–Ozawa) and ASTM E698-based approach to obtain average activation energies. To determine the pre-exponential factor (A), the Coats–Redfern method was applied using multiple kinetic models. The resulting lnA—E_a_ pairs obtained from different models were used to construct lnA = f(E_a_) correlations, enabling the evaluation of the compensation effect and calculation of isokinetic temperatures (T_iso_). **Results**: All three β-lactam antibiotics exhibited consistent kinetic behavior across the applied models, with the F3 reaction model providing the best fit based on R^2^ values. A clear linear relationship between lnA and E_a_ was observed, confirming the presence of an enthalpy–entropy compensation effect. However, significant differences in isokinetic temperatures were obtained indicating variability in kinetic compensation behavior among the studied compounds. **Conclusions**: The thermal degradation of the investigated β-lactam antibiotics follows a consistent kinetic framework, supported by isoconversional and model-fitting approaches. Nevertheless, the absence of a unique isokinetic temperature suggests differences in transition-state stabilization and enthalpy–entropy balance, likely driven by structural variations among the compounds.

## 1. Introduction

The growing interest in the physicochemical characterization of antibiotics highlights the importance of understanding their stability and degradation pathways. In particular, the investigation of thermal degradation kinetics provides critical information regarding the behavior of β-lactam ring, which is known for its pronounced chemical and thermal lability under stress conditions. Therefore, the present study contributes to this field by providing a comparative analysis of cefazolin [1], ceftriaxone [2], and meropenem [3], emphasizing their thermal stability and kinetic parameters. These findings are relevant for pharmaceutical development, storage optimization, and quality control of antibiotic formulations.

β-Lactam antibiotics play a central role in the prevention and treatment of postoperative infectious complications associated with gastrointestinal surgery, especially in patients presenting digestive anastomotic fistulas. These complications are frequently associated with polymicrobial contamination, intra-abdominal sepsis, prolonged hospitalization, and increased morbidity, thus requiring rapid and effective antimicrobial therapy [4,5,6]. Among β-lactam antibiotics, meropenem, ceftriaxone, and cefazolin are commonly used in clinical practice for the management of such infections. Meropenem, a broad-spectrum carbapenem, is preferred in severe or resistant infections due to its potent activity against Gram-negative, Gram-positive, and anaerobic microorganisms. Ceftriaxone, a third-generation cephalosporin, is widely utilized because of its favorable pharmacokinetic properties and efficacy against common enteric pathogens, frequently in combination with anti-anaerobic agents. Cefazolin, a first-generation cephalosporin, remains an important agent for surgical prophylaxis and for the treatment of susceptible infections. Due to their extensive clinical use and therapeutic importance in digestive anastomotic complications, these β-lactam antibiotics represent valuable candidates for investigations concerning their stability, degradation behavior, and pharmaceutical performance [4,5].

Despite their extensive therapeutic use, β-lactam antibiotics represent one of the most clinically important yet chemically unstable classes of antimicrobial agents, mainly due to the high reactivity of the β-lactam ring. Their widespread administration in hospital practice, particularly in severe abdominal and postoperative infections, together with their commercial availability as parenteral formulations, requires strict control of stability during manufacturing, sterilization, storage, and transport conditions. Under thermal or hydrolytic stress, degradation of the β-lactam ring may reduce therapeutic efficacy and promote the formation of degradation products, directly affecting the safety and pharmaceutical performance of these drugs. Although the stability of individual β-lactam antibiotics has been previously investigated, comparative thermoanalytical studies performed under identical experimental conditions remain limited, especially for meropenem, ceftriaxone, and cefazolin. Furthermore, insufficient information is available regarding the relationship between their thermal degradation pathways and the kinetic parameters obtained by non-isothermal methods. Therefore, a systematic comparative evaluation of these antibiotics is necessary in order to better understand the influence of structural differences on their thermal stability and degradation kinetics [6,7].

**Figure 1:**
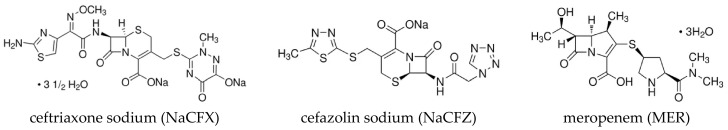
Molecular structures of the investigated β-lactam antibiotics: ceftriaxone sodium, cefazolin sodium, and meropenem.

The thermal stability and degradation behavior of active pharmaceutical ingredients (APIs) are critical parameters influencing their processing, storage, and formulation. In particular, antibiotics such as meropenem, ceftriaxone, and cefazolin exhibit varying degrees of thermal sensitivity due to their distinct chemical structures (Figure 1), including β-lactam rings, which are prone to thermal degradation [8,9].

A comprehensive understanding of their thermal behavior is essential not only for ensuring product quality and safety but also for optimizing manufacturing conditions and predicting shelf life. In this context, thermoanalytical techniques such as thermogravimetric analysis (TG), derivative thermogravimetry (DTG), and differential scanning calorimetry (DSC) are widely employed to investigate mass loss processes, decomposition pathways, and phase transitions.

TG and DTG analyses provide valuable information regarding the number of degradation steps, thermal stability ranges, and maximum decomposition rates, while DSC enables the identification of endothermic and exothermic events associated with melting, crystallization, and chemical decomposition. The combined interpretation of these techniques allows for a more accurate differentiation between physical transitions and chemical degradation processes [10].

Beyond qualitative thermal characterization, the application of non-isothermal kinetic analysis offers deeper insight into the degradation mechanisms of APIs. Methods based on multiple heating rates, such as model-free (isoconversional) approaches, allow for the determination of activation energy as a function of the conversion degree without assuming a predefined reaction model. This is particularly important for complex systems, where degradation often occurs through multi-step processes [11,12].

β-lactam antibiotics, including meropenem, ceftriaxone, and cefazolin, are known for their limited thermal stability due to the inherent reactivity of the β-lactam ring, which undergoes rapid cleavage under thermal stress [7].

Therefore, the aim of this study is to investigate the thermal stability and non-isothermal degradation kinetics of meropenem, ceftriaxone, and cefazolin, using TG/DTG and DSC techniques. The correlation of thermoanalytical data with kinetic parameters enables a detailed understanding of their degradation behavior and provides relevant information for pharmaceutical development and thermal risk assessment.

## 2. Results and Discussion

### 2.1. UATR Analysis

**Figure 2:**
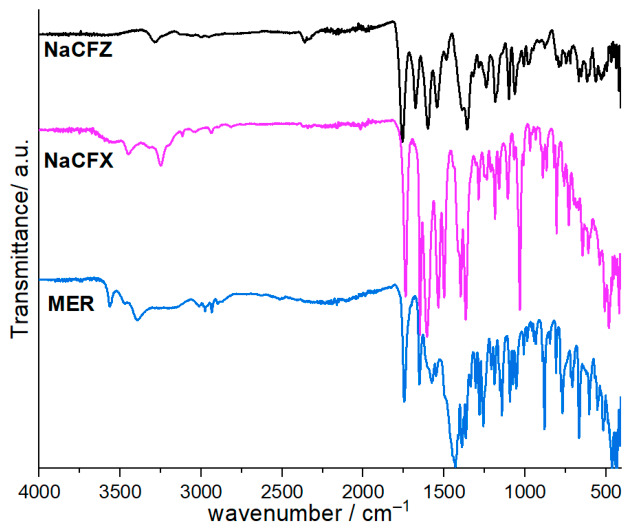
UATR-FTIR spectra of the investigated β-lactam antibiotics: cefazolin sodium, ceftriaxone sodium, and meropenem.

The FTIR spectra of cefazolin sodium, ceftriaxone sodium, and meropenem exhibit characteristic absorption bands consistent with their β-lactam structures and associated functional groups (see Figure 2). All three compounds show broad bands in the region 3400–3200 cm^−1^, which can be attributed to O-H and N-H stretching vibrations, indicating the presence of hydroxyl groups and secondary amines. Weak to moderate bands around 2950–2850 cm^−1^ correspond to aliphatic C-H stretching. A prominent feature in all spectra is the strong absorption in the region 1780–1700 cm^−1^, assigned to the β-lactam C=O stretching vibration. This band is particularly sharp and intense, confirming the integrity of the β-lactam ring. Additional carbonyl contributions from amide and carboxylate groups appear in the 1700–1600 cm^−1^ region. In ceftriaxone and meropenem, the complexity and splitting of these bands reflect multiple carbonyl groups. Bands observed near 1600–1500 cm^−1^ are attributed to N-H bending and C=C stretching vibrations within aromatic or heterocyclic rings. The spectra of ceftriaxone and cefazolin, which contain more complex ring systems, display more pronounced features in this region. In the fingerprint region (1500–500 cm^−1^), all compounds show numerous intense and overlapping bands. These are associated with C-N, C-O, and C-S stretching vibrations, as well as ring deformations. Ceftriaxone exhibits particularly dense and sharp peaks in this region, indicating a more complex molecular structure with multiple functional groups. Meropenem shows strong absorptions around 1200–1000 cm^−1^, characteristic of C-N and C-O stretching in carbapenem systems. Overall, the FTIR spectra confirm the presence of key functional groups typical for β-lactam antibiotics, including lactam carbonyls, amide linkages, and heterocyclic ring structures, with spectral differences reflecting the structural complexity of each compound [13,14,15].

### 2.2. Thermoanalytical Profile

**Figure 3:**
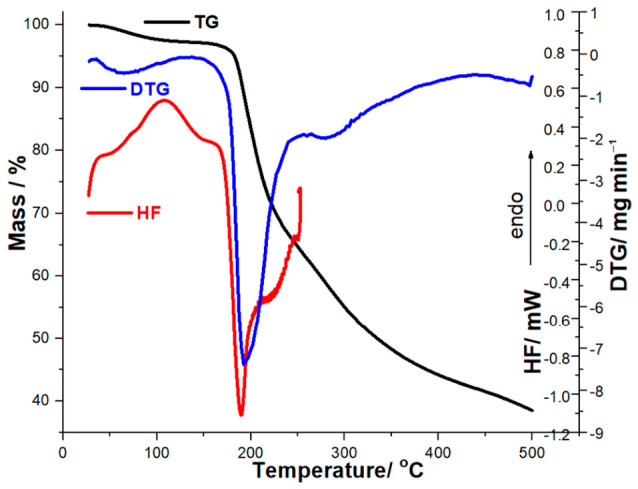
Thermoanalytical curves for cefazolin sodium recorded at 10 °C min^−1^.

Sodium cefazolin exhibits a complex thermal profile, as evidenced by TG, DTG, and DSC data. An initial minor mass loss below 150 °C, observed in the TG curve, correlates with a weak endothermic DSC signal, between approximately 40 and 120 °C, with a maximum around 90 °C, suggesting the removal of adsorbed or loosely bound water indicating that the sample is present in a hydrated form (see Figure 3). The broad nature of this signal suggests gradual dehydration rather than a well-defined phase transition. The main degradation process occurs between 180 °C and 275 °C, with a DTG peak at 193 °C, confirming that this is the maximum rate of mass loss and the main thermal degradation stage (see Table 1).

Concomitantly, the HF curve exhibits a strong exothermic peak in the same temperature region, indicating that the decomposition process is predominantly exothermic. This thermal event is associated with bond dissociation and structural collapse of the antibiotic molecule indicating that thermal decomposition overlaps or follows closely after any possible melting process, which is not clearly distinguishable as a separate peak [16]. Beyond 230 °C, the TG curve shows a gradual and continuous mass loss up to 500 °C, which can be attributed to further degradation and carbonization of the residual organic matrix. The DTG signal in this region is broad and less intense, suggesting slower, overlapping secondary decomposition processes. The absence of sharp peaks indicates that no well-defined additional decomposition steps occur, but rather a progressive breakdown of remaining fragments. The significant residual mass observed above 500 °C (Δm ≈ 40%) for cefazolin sodium is attributed to the formation of thermally stable inorganic species, such as sodium carbonate and sodium sulfate, along with carbonaceous residues. These products obtained from the complete degradation of the organic structure and subsequent recombination and oxidation of sodium- and sulfur-containing fragments. The DSC baseline in this region may show broad or overlapping signals, confirming the complexity of the degradation pathway. Overall, sodium cefazolin demonstrates limited thermal stability, with its main degradation occurring around 190–200 °C (see Table 1). The combined TG-DTG-HF analysis confirms a dehydration stage followed by a dominant endothermic decomposition process and subsequent gradual degradation at higher temperatures.

**Figure 4:**
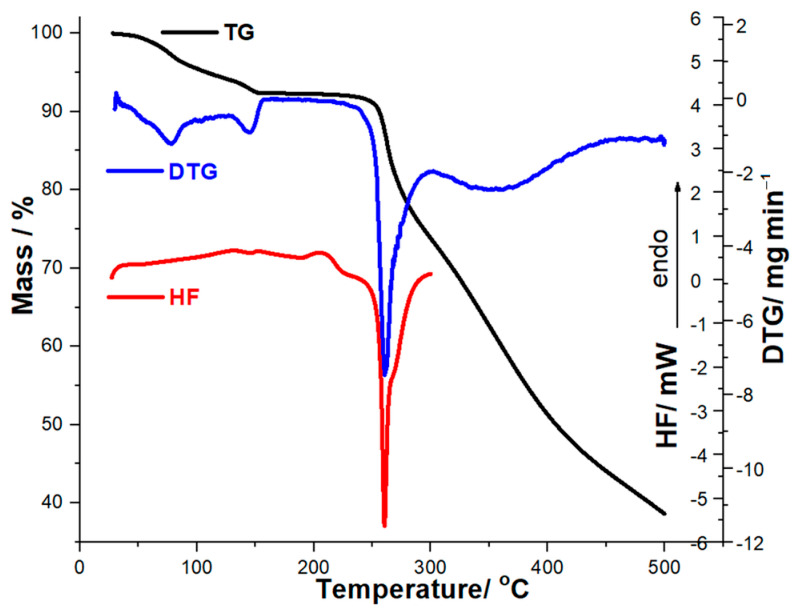
Thermoanalytical curves for ceftriaxone sodium recorded at 10 °C min^−1^.

The second active substance investigated using thermoanalytical techniques (TG/DTG/HF) is ceftriaxone sodium. The TG curve reveals an initial minor mass loss below 160 °C, attributed to the removal of physically adsorbed and/or crystallization water, indicating the hydrated nature of the compound (see Figure 4). A major mass loss step occurs in the temperature range of 230–300 °C, accompanied by a pronounced peak in the DTG curve (260 °C) and a strong exothermic effect in the HF signal (260 °C). This stage is associated with the thermal decomposition of the ceftriaxone structure. Above 300 °C, a continuous mass decrease is observed, corresponding to the further decomposition and carbonization of intermediate products. At temperatures above 500 °C, the mass represents a residual fraction likely composed of inorganic sodium-containing species and carbonaceous material. Overall, sodium ceftriaxone exhibits a multistep thermal degradation process, with the main decomposition event occurring around 230–300 °C (see Table 1).

**Figure 5:**
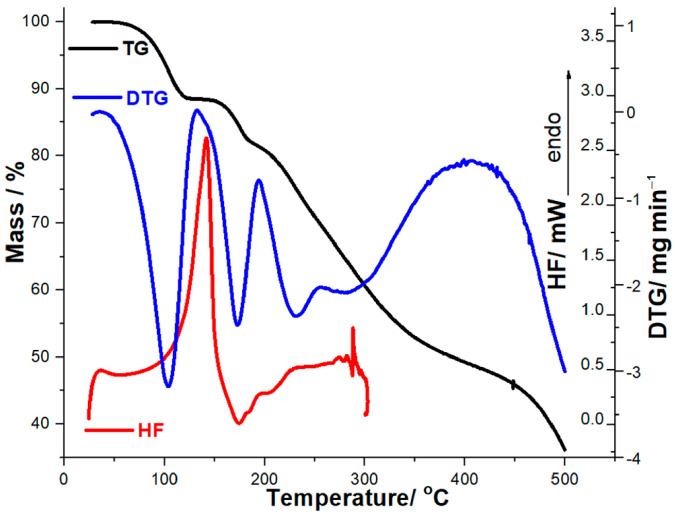
Thermoanalytical curves for meropenem recorded at 10 °C min^−1^.

The thermoanalytical curves for meropenem reveal a complex, multistep thermal degradation process characteristic of solid-state pharmaceuticals (see Figure 5). The TG curve shows an initial mass stability up to approximately 80–100 °C, suggesting the absence of significant moisture or solvent content, or only minor physically adsorbed water. A first noticeable mass loss occurs in the range of 100–160 °C, corresponding to the dehydration stage. The percentage of mass loss recorded on the TG curve (11.5%) agrees with the theoretical value calculated (12%) for meropenem containing three molecules of hydration water. This step is relatively limited in magnitude and is associated with the initial structural destabilization of the molecule. The DTG curve highlights multiple overlapping peaks, confirming that the degradation process is not a single-step reaction. The first prominent DTG peak around 120–140 °C corresponds to the primary onset of decomposition. This is followed by additional peaks in the 170–250 °C range, indicating consecutive degradation steps, likely involving overlapping structural decomposition processes characteristic of β-lactam antibiotics and subsequent breakdown of the molecular structure. The HF signal provides further insight into the energetic aspects of these processes. The presence of both endothermic and exothermic effects suggests competing phenomena. The initial region may include endothermic contributions (structural rearrangements and/or desolvation phenomena), followed by more pronounced exothermic effects associated with secondary decomposition processes associated with the progressive destabilization of the molecular structure. The main exothermic event appears to coincide with the most intense DTG peak, indicating that the principal mass loss stage is energetically significant. At higher temperatures (above 300 °C), the TG curve shows a continuous mass decrease extending up to 500 °C, corresponding to the progressive decomposition of intermediate residues and eventual formation of a carbonaceous char. The broad DTG signal in this region indicates slower, diffusion or structure-controlled processes rather than well-defined reaction steps.

### 2.3. The Kinetic Analysis

An isoconversional kinetic approach was adopted to investigate the thermal degradation behavior of the three active substances. Such methods are particularly suitable for complex systems, as they provide model-free evaluation of the apparent activation energy. The ASTM E698 [17], Friedman [18], and Flynn–Wall–Ozawa [19,20] methods were applied to ensure the robustness and consistency of the kinetic parameters. These approaches allow the assessment of the variation in activation energy as a function of the conversion degree, offering valuable insights into the degradation mechanism. The combined use of differential and integral methods ensures a comprehensive kinetic analysis and improves the reliability of the obtained results (see Table 2).

**Figure 6:**
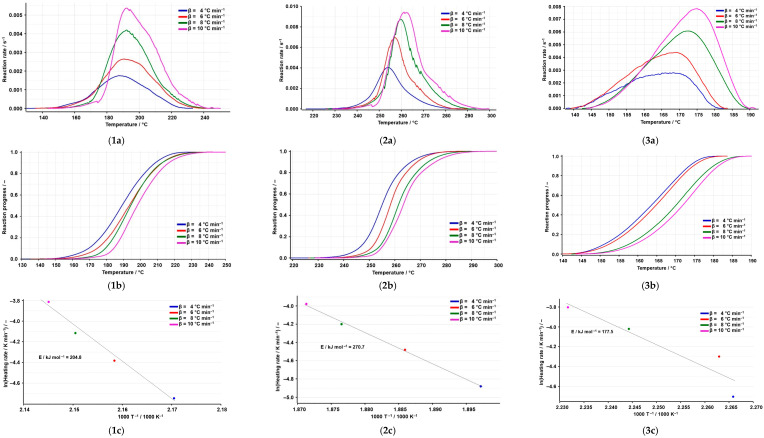

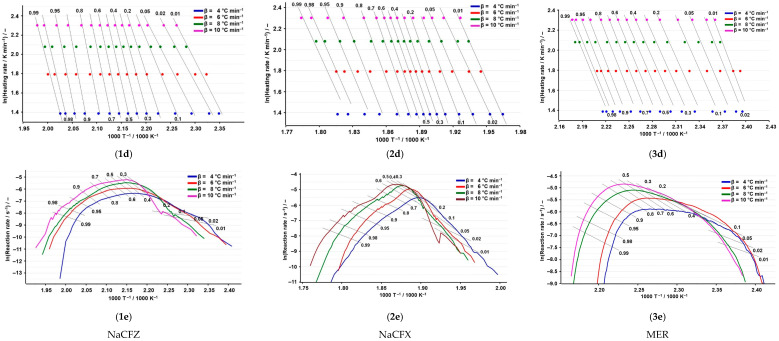
Kinetic analysis of (**1**) NaCFZ, (**2**) NaCFX, and (**3**) MER at different heating rates (4, 6, 8, and 10 °C min^−1^). (**a**) reaction rate vs temperature; (**b**) conversion degree as a function of temperature; (**c**) ASTM E698 plots used for activation energy determination; (**d**) Flynn–Wall–Ozawa (FWO) isoconversional plots; (**e**) linear plotting of Friedman method.

The kinetic behavior of cefazolin sodium was evaluated using multiple heating rates (4, 6, 8, and 10 °C·min^−1^), revealing a complex thermally activated degradation process. The reaction rate curves (dα/dt vs. T) show a clear shift in the maximum reaction rate toward higher temperatures with increasing heating rate. This behavior confirms that the degradation process is kinetically controlled and follows typical non-isothermal reaction kinetics (see Figure 6(1a)). Additionally, the increase in peak intensity at higher heating rates suggests accelerated degradation under faster thermal conditions. The conversion curves (α vs. T) exhibit sigmoidal profiles, indicating a gradual transition from initial stability to rapid degradation, followed by completion (see Figure 6(1b)). The shift in these curves to higher temperatures with increasing heating rate further supports the thermal dependence of the process. The plots of ln(β) versus 1/T at constant conversion (α) show good linearity across a wide conversion range (α ≈ 0.1–0.9), indicating that the applied FWO isoconversional method is appropriate for describing the degradation kinetics (see Figure 6(1d)). However, slight deviations at very low (α < 0.1) and very high conversion degrees (α > 0.90) suggest the presence of secondary processes, such as: structural relaxation and/or final-stage decomposition and residue formation. The variation in slopes across different α values demonstrates that the activation energy is conversion-dependent, confirming a multi-step degradation mechanism rather than a single elementary reaction. The linear dependence of ln(β/T^2^) on 1/T_peak_ obtained for cefazolin, in accordance with the ASTM E698 method (Figure 6(1d)), confirms the applicability of the Arrhenius approach and yields an activation energy of approximately 204.8 kJ·mol^−1^, indicating a relatively high thermal stability and a degradation process governed by a single dominant reaction step.

For ceftriaxone sodium, the activation energy determined using the ASTM E698 method is approximately 270.67 kJ·mol^−1^, as obtained from the linear fit of the logarithmic heating rate versus reciprocal temperature. The good linearity of the plot indicates a consistent kinetic behavior over the selected temperature range. However, as a single-point method, ASTM provides only an average activation energy and does not reflect potential variations during the degradation process. The Flynn–Wall–Ozawa plots corresponding to different degrees of conversion (α = 0.01–0.99) exhibit a series of nearly parallel straight lines, suggesting a relatively uniform degradation mechanism over a broad conversion range. The slight deviations from parallelism observed at higher conversion degrees may indicate changes in the reaction mechanism or the contribution of secondary processes. Overall, the FWO method confirms the multistep nature of ceftriaxone thermal degradation. The Friedman differential analysis reveals a noticeable variation in the apparent activation energy as a function of conversion. The activation energy initially increases, reaching a maximum at intermediate conversion levels (α ≈ 0.3–0.6) followed by a gradual decrease at higher conversion degrees. This behavior suggests that the degradation process involves multiple overlapping thermal events and intermediate stages rather than a single elementary reaction mechanism.

The thermal degradation of meropenem was investigated under non-isothermal conditions at different heating rates, revealing a strong dependence of the kinetic parameters on experimental conditions. The derivative conversion curves (dα/dT) show a systematic shift in the peak temperature (T_p_) toward higher values with increasing heating rate (β), indicating kinetic control of the process and the absence of instantaneous thermal equilibrium. The α = f(T) curves exhibit a sigmoidal profile typical of solid-state degradation, suggesting sequential initiation, propagation, and termination steps. The observed shift toward higher temperatures with increasing β indicates a thermal lag effect associated with heat transfer limitations and reduced reaction time. The activation energy determined by the ASTM method is approximately 177.5 kJ/mol, indicating high thermal stability of meropenem. This value reflects the involvement of strong bond cleavage and a significant energy requirement for degradation. The increase in T_p_ with β further confirms a kinetically controlled mechanism and suggests a complex, likely multistep degradation pathway typical of β-lactam antibiotics. Isoconversional analysis shows that the activation energy depends moderately on conversion, ranging from 138 to 157 kJ/mol (FWO) and from 114 to 180 kJ/mol (FR). The mean values are 146.3 ± 32.8 kJ/mol (FWO) and 153.7 ± 36.6 kJ/mol (FR), indicating good agreement between methods, despite the relatively large associated standard deviations. The dependence of E_a_ on conversion confirms a multistep degradation mechanism. At low conversions (α < 0.2), lower activation energies correspond to the onset of thermally less stable structural transformations occurring during the early stages of degradation. At intermediate conversions (α = 0.3–0.7), E_a_ remains relatively constant (especially for FWO), indicating a dominant kinetic regime. The Friedman method shows higher dispersion, reflecting sensitivity to experimental noise and possible overlapping processes. At high conversions (α > 0.8), a slight decrease in *E_a_* is observed, likely due to diffusion limitations and the degradation of thermally stable residues. Such behavior is typical for solid-state decomposition systems. Compared with other antibiotics, meropenem exhibits intermediate activation energy values, indicating moderate thermal stability—lower than ceftriaxone sodium but comparable to or slightly higher than cefazolin sodium, depending on conversion range. These observations are consistent with a complex degradation pathway involving initial bond cleavage, followed by major structural decomposition and subsequent secondary reactions of the degradation products. The non-uniform DTG profile and the presence of multiple thermal events support the conclusion that the process cannot be described by a single kinetic model, in agreement with the isoconversional analysis.

**Figure 7:**
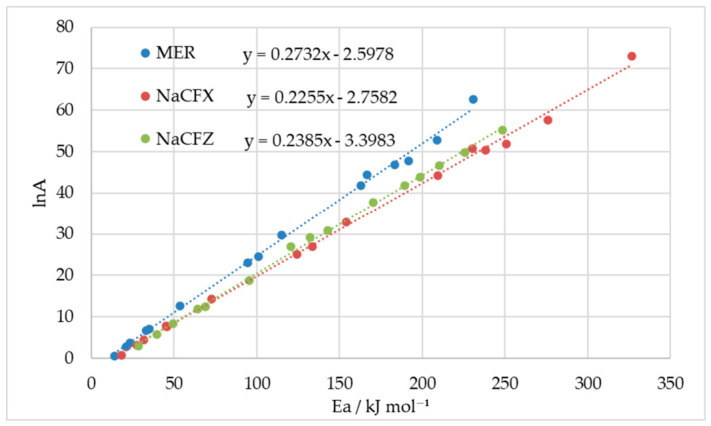
Linear correlation between lnA and activation energy (E_a_) for three β-lactam antibiotics, showing the compensation effect in kinetic parameters.

The Coats–Redfern method [21,22] was applied using multiple kinetic models to generate (E_a_, lnA) data pairs, enabling the evaluation of the kinetic compensation effect for all three β-lactam compounds. A total of fifteen kinetic models in their integral form were applied to all four heating rates of the studied compounds. The best fit (highest R^2^) was consistently obtained with the F3 model for all three active substances (see Table 3). A clear linear correlation between lnA and Ea was observed for all active substances, confirming the presence of a kinetic compensation effect [23,24]. The high correlation coefficients indicate a strong interdependence between the activation energy and the pre-exponential factor for all three beta-lactams. The kinetic compensation effect was evaluated based on the relationship between the Arrhenius parameters obtained from the applied kinetic models. The apparent activation energy (E_a_) and pre-exponential factor (A) listed in Table 3 were used to construct lnA versus E_a_ plots for each beta-lactam antibiotics (MER, NaCFX, and NaCFZ). A linear regression analysis was performed according to the compensation equation lnA = a∙E_a_ + b, where *a* represents the slope and b the intercept, for the calculation of isokinetic temperature values (T_iso_) which is defined as the temperature at which all reactions in a series have equal rate constants, evidencing enthalpy–entropy compensation. The isokinetic temperature was calculated from the slope using the relation T_iso_ = 1/(a∙R), where R is the universal gas constant [23]. The presence of a linear correlation between lnA and E_a_ was taken as evidence of the kinetic compensation effect. For the three active substances subjected to kinetic analysis, the lnA = f(E_a)_ compensation plots are shown in Figure 7, whereas the corresponding Arrhenius parameters, together with the derived isokinetic temperature (T_iso_), are compiled in Table 4.

Analyzing the obtained data, it can be observed that there is no single isokinetic temperature characteristic of the three antibiotics. Although all three active substances exhibit the highest R^2^ value for the F3 model, suggesting a high probability that they follow the similar kinetic behavior under the selected experimental conditions, the considerable differences in T_iso_ values may be attributed to significant variations in transition-state stabilization due to substituents that differ both in number and structure. Thus, it can be stated that in the case of NaCFX, which exhibits the highest T_iso_, the system is kinetically more “rigid”, and the enthalpy–entropy compensation is more temperature-dependent, in contrast to MER, which shows the lowest T_iso_ and therefore a weaker compensation effect.

From a pharmaceutical perspective, the obtained kinetic parameters should be interpreted primarily as comparative indicators of thermal stability and temperature sensitivity rather than direct predictors of shelf life under conventional storage conditions. Since the experiments were performed under non-isothermal conditions and at elevated temperatures, direct extrapolation to room-temperature stability may not accurately reflect real-time degradation behavior. Nevertheless, the data provide useful information for identifying temperature-sensitive compounds and for defining suitable conditions during pharmaceutical processing, drying, sterilization, transportation, and storage.

In practical pharmaceutical settings, β-lactam antibiotics may be exposed to elevated temperatures during several technological and handling stages, including spray drying, lyophilization, sterilization procedures, granulation, accelerated stability testing, transportation, and improper storage conditions. In hospital environments, reconstituted injectable formulations may also experience temporary temperature fluctuations during preparation or administration. Although the degradation temperatures identified in this study are significantly higher than conventional storage temperatures, the obtained kinetic parameters provide valuable comparative information regarding the intrinsic thermal sensitivity of the investigated compounds. Such data are important for predicting the relative susceptibility of these antibiotics to thermal stress, selecting appropriate formulation and processing conditions, and minimizing degradation during pharmaceutical manufacturing and storage [26].

It should be emphasized that the kinetic parameters obtained from non-isothermal thermal analysis describe the global thermal degradation behavior of the investigated compounds and should not be interpreted as direct evidence of specific bond cleavage events or individual degradation pathways. The observed degradation processes likely involve multiple overlapping reactions and intermediate stages characteristic of complex solid-state decomposition systems. Although β-lactam antibiotics are generally known to exhibit limited thermal stability due to the reactive nature of the β-lactam ring, confirmation of specific degradation mechanisms would require complementary analytical techniques such as evolved gas analysis, TGA-FTIR, or LC-MS characterization of degradation products.

## 3. Materials and Methods

The beta-lactam active substances: cefazolin sodium (NaCFZ, ID: 27164-46-1), ceftriaxone sodium (NaCFX, ID: Y0000138), and meropenem (MER, ID: M0215000) were purchased from Sigma-Aldrich (St. Louis, MO, USA). Their purity complied with the specifications of the European Pharmacopoeia (EP) Reference Standards. All compounds were stored in tightly closed vials according to the manufacturer’s recommendations and were used as received, without any further preparation.

The FTIR spectra of the three investigated β-lactam antibiotics were recorded using an IRSpirit Fourier Transform Infrared spectrophotometer (Shimadzu, Kyoto, Japan). Measurements were performed over the spectral range of 4000–400 cm^−1^, each spectrum being obtained by averaging 32 scans at a resolution of 2 cm^−1^.

Thermogravimetric analyses (TG/DTG) were performed using a NETZSCH thermobalance (NETZSCH, Selb, Germany) under a dynamic air atmosphere (100 mL·min^−1^), in open aluminium crucibles (85 μL). The sample mass ranged between 5 and 10 mg, ensuring reliable thermal response and accurate monitoring of mass changes during the measurements. The synthetic air consisted of 20% oxygen (O_2_) and 80% nitrogen (N_2_). Temperature calibration was carried out using certified reference materials with well-defined melting points (e.g., indium and zinc). The experiments were conducted under non-isothermal conditions at four heating rates (β = 4, 6, 8, and 10 °C·min^−1^), from room temperature up to 500 °C.

Differential scanning calorimetry (DSC) measurements were performed using a NETZSCH DSC 204 F1 Phoenix instrument (NETZSCH, Selb, Germany). Temperature calibration was achieved using high-purity standards (indium, tin, and zinc) with certified melting points, while enthalpy calibration was based on the known heat of fusion of indium. Approximately 5.0 mg of sample was placed in a pierced aluminum crucible (40 μL) and heated from 25 to 300 °C at a heating rate of 10 °C·min^−1^ under a nitrogen atmosphere (20 mL min^−1^), using an empty aluminum pan as reference. The heat flow (HF, mW) data were obtained directly from the DSC measurements. All experiments were performed in triplicate to ensure reproducibility.

The kinetic analysis of the main decomposition stage of the three investigated β-lactam antibiotics—cefazolin, ceftriaxone, and meropenem—was performed using the ASTM E698, Friedman (FR), and Flynn–Wall–Ozawa (FWO) methods, as implemented in AKTS Thermokinetics software (Version 4.46, AKTS AG TechnoArk, Siders, Switzerland). The theoretical background and significance of isoconversional kinetic approaches have been extensively reported in the literature [27].

## 4. Conclusions

This study provided a comparative thermoanalytical and kinetic evaluation of three representative β-lactam antibiotics: cefazolin sodium, ceftriaxone sodium, and meropenem, using TG/DTG/DSC techniques combined with non-isothermal kinetic approaches. All compounds exhibited multistep thermal degradation processes preceded, in some cases, by dehydration phenomena associated with hydrated solid forms. The principal decomposition stage was strongly influenced by molecular structure, with ceftriaxone sodium showing the highest thermal resistance, followed by cefazolin sodium and meropenem, according to the E_a_ values.

The activation energies obtained by the ASTM E698, Flynn–Wall–Ozawa and Friedman methods confirmed that degradation does not proceed through a simple single-step pathway, but rather through overlapping processes whose apparent energy barrier varies with conversion degree. Nevertheless, the good agreement between the applied methods supports the robustness of the kinetic treatment.

Among the tested solid-state kinetic models, the F3 model gave the best correlation coefficients for all three antibiotics, suggesting that despite structural differences, their dominant decomposition stage may follow a similar reaction framework under the selected experimental conditions.

A pronounced linear relationship between lnA and E_a_ was observed for each compound, confirming the presence of a kinetic compensation effect. However, the calculated isokinetic temperatures differed significantly (MER < NaCFZ < NaCFX), indicating that no universal isokinetic temperature exists for the studied β-lactams. This finding suggests that substituent effects and specific molecular architecture strongly influence transition-state stabilization and the enthalpy–entropy balance during degradation.

Overall, these results provide useful insight into the thermal behavior and degradation kinetics of β-lactam antibiotics, with practical relevance for formulation development, processing conditions, storage optimization, and prediction of thermal stability in pharmaceutical applications. The isokinetic temperatures determined for NaCFX (260.0 °C), NaCFZ (190.0 °C), and MER (167.3 °C) indicate the presence of enthalpy–entropy compensation across all systems, although the significant variation in T_iso_ values suggests differences in the underlying reaction mechanisms and transition-state stabilization.

## Figures and Tables

**Table 1:** Results of the thermal investigations for the three active pharmaceutical ingredients.

API	TG		Δm/%	DTG	DSC
	T_onset_/°C	T_offset_/°C		T_peak_/°C	T_peak_/°C
NaCFZ	50	150	3	65	90 (endo); 170 (endo)
	180	275	36	193	
	275	500	20.5	307	
MER	50	132	11.5	104	142 (endo)
	135	194	7.09	173	
	194	406	32.5	231; 283	
NaCFX	35	150	7.7	80; 145	261 (exo)
	230	250	18.4	260	
	250	500	35.2	363	

**Table 2:** Activation energy as a function of the degree of conversion (α) for three beta-lactam antibiotics, determined using the Flynn–Wall–Ozawa (FWO) and Friedman (FR) methods.

Ea (kJ∙mol^–1^) vs. α for						
	Sodium Cefazolin		Sodium Ceftriaxone		Meropenem	
α	FWO	FR	FWO	FR	FWO	FR
0.05	79.0 ± 0.7	136.9 ± 1.2	198.1 ± 0.5	304.0 ± 20.1	143.9 ± 7.6	114.3 ± 4.8
0.10	98.7 ± 0.7	174.1 ± 0.9	221.3 ± 1.8	322.9 ± 6.0	138.4 ± 6.6	128.3 ± 8.1
0.15	112.9 ± 0.7	187.2 ± 1.1	234.2 ± 1.9	310.8 ± 3.3	137.0 ± 6.6	131.6 ± 6.7
0.20	125.6 ± 0.9	203.5 ± 1.6	241.2 ± 1.5	296.3 ± 2.4	137.1 ± 6.6	136.9 ± 7.1
0.25	137.9 ± 1.1	211.3 ± 2.5	244.2 ± 1.0	272.6 ± 4.8	137.2 ± 6.6	137.9 ± 7.5
0.30	149.2 ± 1.4	217.3 ± 3.0	244.3 ± 0.6	262.7 ± 5.0	137.6 ± 6.5	141.5 ± 5.7
0.35	159.4 ± 1.8	218.2 ± 4.1	243.4 ± 0.4	253.1 ± 4.8	138.4 ± 6.5	147.8 ± 7.0
0.40	167.9 ± 2.5	219.3 ± 6.2	242.4 ± 0.3	238.8 ± 7.2	139.6 ± 6.6	154.6 ± 6.8
0.45	174.4 ± 3.3	215.9 ± 9.1	242.1 ± 0.3	236.7 ± 6.9	141.4 ± 6.7	161.2 ± 7.1
0.50	179.5 ± 4.8	214.0 ± 11.9	242.5 ± 0.5	234.8 ± 6.7	143.8 ± 6.9	169.1 ± 7.7
0.55	184.4 ± 6.0	212.8 ± 14.4	243.0 ± 0.7	232.3 ± 6.3	146.6 ± 7.2	176.6 ± 7.8
0.60	188.7 ± 7.3	213.1 ± 15.4	243.2 ± 1.1	226.9 ± 6.6	149.3 ± 7.5	177.7 ± 8.4
0.65	191.5 ± 8.7	216.9 ± 16.0	242.4 ± 1.5	229.3 ± 3.7	152.1 ± 7.9	180.2 ± 9.1
0.70	194.9 ± 10.5	224.3 ± 16.9	240.7 ± 2.0	210.0 ± 6.9	154.4 ± 8.2	177.7 ± 10.2
0.75	200.2 ± 12.3	230.9 ± 17.8	237.2 ± 2.3	201.5 ± 4.8	156.2 ± 8.6	173.2 ± 11.0
0.80	205.0 ± 14.5	232.9 ± 18.6	231.3 ± 2.4	180.0 ± 8.6	157.2 ± 8.9	165.8 ± 11.3
0.85	210.8 ± 15.5	220.1 ± 14.2	222.1 ± 2.9	179.7 ± 13.5	157.2 ± 9.1	155.4 ± 11.3
0.90	214.2 ± 12.3	207.8 ± 4.2	212.9 ± 4.1	180.8 ± 21.4	156.9 ± 8.8	149.0 ± 9.8
0.95	202.7 ± 6.1	147.4 ± 4.9	201.2 ± 7.1	185.4 ± 26.1	155.3 ± 8.4	140.7 ± 8.5
$\bar{E}$_a_ (kJ∙mol^–1^)	167.2 ± 7.9	205.5 ± 46.8	233.0 ± 10.3	239.9 ± 47.3	146.3 ± 32.8	153.7 ± 36.6

**Table 3:** Kinetic model fitting results for the conversion degree (α) using various solid-state reaction models. The integral forms of the kinetic functions, g(α), are listed for each model, including reaction-order models (F1–F3), diffusion models (D1–D4), nucleation and growth models (A2–A4), contracting geometry models (R2–R3), and power-law models (P2–P4). The apparent activation energy (E_a_ ± SD), pre-exponential factor (lnA ± SD), and coefficient of determination (R^2^) are reported for three methods: MER, NaCFX, and NaCFZ [25].

	Model Name	g(α) (Integral Form)	MER			NaCFX			NaCFZ		
			Ea ± SD	lnA ± SD	R^2^	Ea ± SD	lnA ± SD	R^2^	Ea ± SD	lnA ± SD	R^2^
F1	First-order reaction	−ln(1 − α)	114.8 ± 16.0	29.7 ± 4.7	0.966	153.9 ± 16.4	32.9 ± 4.0	0.904	85.4 ± 4.9	19.9 ± 1.6	0.947
F2	Second-order reaction	(1/(1 − α))−1	166.4 ± 23.8	44.5 ± 6.8	0.994	230.5 ± 25.0	50.8 ± 5.9	0.966	128.4 ± 9.8	31.6 ± 2.8	0.989
F3	Third-order reaction	((1 − α)^−2^ − 1)/2	230.6 ± 33.7	62.6 ± 9.5	0.996	326.5 ± 35.9	73.0 ± 8.2	0.988	182.4 ± 16.3	46.2 ± 4.4	0.999
D1	One-dimensional diffusion (parabolic law)	α^2^	163.0 ± 21.5	41.8 ± 6.1	0.909	209.5 ± 21.1	44.3 ± 5.0	0.808	118.2 ± 4.2	27.3 ± 1.3	0.869
D2	Two-dimensional diffusion	(1 − α)ln(1 − α) + α	183.3 ± 24.2	46.9 ± 6.9	0.931	238.5 ± 24.1	50.4 ± 5.7	0.842	134.5 ± 5.5	31.1 ± 1.7	0.898
D3	Three-dimensional diffusion (Jander equation)	(1 − (1 − α)^1/3^)^2^	209.2 ± 27.9	52.8 ± 7.9	0.952	276.1 ± 28.2	57.7 ± 6.5	0.880	155.7 ± 7.4	35.3 ± 2.2	0.929
D4	Three-dimensional diffusion (Ginstling–Brounshtein)	1 − 2α/3 −(1 − α)^2/3^	191.8 ± 25.4	47.8 ± 7.2	0.939	250.8 ± 25.5	51.8 ± 5.9	0.856	141.5 ± 6.1	31.5 ± 1.8	0.909
A2	Avrami–Erofeev (nucleation and growth, n = 2)	[−ln(1 − α)]^1/2^	53.7 ± 8.0	12.7 ± 2.6	0.961	72.4 ± 8.2	14.2 ± 2.3	0.893	38.7 ± 2.4	7.5 ± 1.0	0.935
A3	Avrami–Erofeev (nucleation and growth, n = 3)	[−ln(1 − α)]^1/3^	33.3 ± 5.3	6.8 ± 1.9	0.955	45.3 ± 5.4	7.8 ± 1.7	0.879	23.1 ± 1.6	3.2 ± 0.8	0.920
A4	Avrami–Erofeev (nucleation and growth, n = 4)	[−ln(1 − α)]^1/4^	23.1 ± 4.0	3.7 ± 1.6	0.947	31.7 ± 4.1	4.5 ± 1.4	0.863	15.4 ± 1.2	0.8 ± 0.7	0.899
R2	Contracting area	1 − (1 − α)^1/2^	94.5 ± 13.0	23.1 ± 3.9	0.938	124.3 ± 13.1	25.2 ± 3.3	0.854	68.7 ± 3.2	14.6 ± 1.2	0.906
R3	Contracting volume	1 − (1 − α)^1/3^	100.9 ± 14.0	24.6 ± 4.2	0.949	133.5 ± 14.1	27.0 ± 3.5	0.872	73.9 ± 3.7	15.6 ± 1.3	0.921
P2	Power law (n = 2)	α^1/2^	35.2 ± 5.3	7.1 ± 1.9	0.880	45.6 ± 5.2	7.6 ± 1.6	0.759	23.6 ± 1.0	3.0 ± 0.6	0.806
P3	Power law (n = 3)	α^1/3^	21.0 ± 3.6	2.9 ± 1.5	0.853	27.4 ± 3.5	3.2 ± 1.3	0.718	13.0 ± 0.7	−0.1 ± 0.6	0.740
P4	Power law (n = 4)	α^1/4^	13.9 ± 2.7	0.6 ± 1.3	0.816	18.3 ± 2.6	0.8 ± 1.1	0.667	7.8 ± 0.5	−1.8 ± 0.5	0.641

**Table 4:** Kinetic model fitting results for the conversion degree (α) using various solid-state reaction models. The integral forms of the kinetic functions.

API	a	b	T_iso_ (°C)	R^2^
NaCFX	0.2255	−2.7582	260.0	0.9979
NaCFZ	0.2385	−3.3983	190.0	0.9986
MER	0.2732	−2.5978	167.3	0.9973

## Data Availability

The original contributions presented in this study are included in the article. Further inquiries can be directed to the corresponding authors.
